# Supplementary material for: HIVprotI: an integrated web based platform for prediction and design of HIV proteins inhibitors
Source: J Cheminform. 2018 Mar 9;10:12. doi: 10.1186/s13321-018-0266-y (PMC5845081; doi:10.1186/s13321-018-0266-y)
Supplement: Supplementary file 2 — Additional file 2. Source code of HIVProtI web server. [file 13321_2018_266_MOESM2_ESM.zip › HIVprotI_Source-code/hivprotI/weka-3-6-6/doc/weka/gui/SysErrLog.html]

SysErrLog


---


|  |  |  |
| --- | --- | --- |
| **Overview** \| **Package** \| **Class** \| **Tree** \| **Deprecated** \| **Index** \| **Help** \| **Weka's home** | |  |
| **PREV CLASS**   **NEXT CLASS** | **FRAMES**    **NO FRAMES**     **All Classes** |  |
| SUMMARY: NESTED \| FIELD \| CONSTR \| METHOD | DETAIL: FIELD \| CONSTR \| METHOD |  |


---


## weka.gui Class SysErrLog

```
java.lang.Object
  weka.gui.SysErrLog
```

**All Implemented Interfaces:**: Logger

---

``` public class SysErrLog extends java.lang.Object implements Logger ```

This Logger just sends messages to System.err.

**Version:**
:   $Revision: 7059 $

**Author:**
:   Len Trigg (trigg@cs.waikato.ac.nz)

---

**Constructor Summary**

`SysErrLog()`


| **Method Summary** | |
| --- | --- |
| `void` | `logMessage(java.lang.String message)`             Sends the supplied message to the log area. |
| `void` | `statusMessage(java.lang.String message)`             Sends the supplied message to the status line. |

**Methods inherited from class java.lang.Object**

`equals, getClass, hashCode, notify, notifyAll, toString, wait, wait, wait`

**Constructor Detail**

### SysErrLog

```
public SysErrLog()
```


**Method Detail**

### logMessage

```
public void logMessage(java.lang.String message)
```

:   Sends the supplied message to the log area. The current timestamp will
    be prepended.

    :   **Specified by:**: `logMessage` in interface `Logger`
    :   **Parameters:**: `message` - a value of type 'String'

---


### statusMessage

```
public void statusMessage(java.lang.String message)
```

:   Sends the supplied message to the status line.

    :   **Specified by:**: `statusMessage` in interface `Logger`
    :   **Parameters:**: `message` - the status message


---


|  |  |  |
| --- | --- | --- |
| **Overview** \| **Package** \| **Class** \| **Tree** \| **Deprecated** \| **Index** \| **Help** \| **Weka's home** | |  |
| **PREV CLASS**   **NEXT CLASS** | **FRAMES**    **NO FRAMES**     **All Classes** |  |
| SUMMARY: NESTED \| FIELD \| CONSTR \| METHOD | DETAIL: FIELD \| CONSTR \| METHOD |  |


---
